# Tripping the light fantastic in membrane redox biology: linking dynamic structures to function in ER electron transfer chains

**DOI:** 10.1111/febs.14757

**Published:** 2019-01-30

**Authors:** Tobias M. Hedison, Nigel S. Scrutton

**Affiliations:** ^1^ Manchester Institute of Biotechnology and School of Chemistry University of Manchester UK

**Keywords:** cytochrome P450, cytochrome P450 reductase, electron transfer chemistry, membrane protein, protein domain dynamics

## Abstract

How the dynamics of proteins assist catalysis is a contemporary issue in enzymology. In particular, this holds true for membrane‐bound enzymes, where multiple structural, spectroscopic and biochemical approaches are needed to build up a comprehensive picture of how dynamics influence enzyme reaction cycles. Of note are the recent studies of cytochrome P450 reductases (CPR)–P450 (CYP) endoplasmic reticulum redox chains, showing the relationship between dynamics and electron flow through flavin and haem redox centres and the impact this has on monooxygenation chemistry. These studies have led to deeper understanding of mechanisms of electron flow, including the timing and control of electron delivery to protein‐bound cofactors needed to facilitate CYP‐catalysed reactions. Individual and multiple component systems have been used to capture biochemical behaviour and these have led to the emergence of more integrated models of catalysis. Crucially, the effects of membrane environment and composition on reaction cycle chemistry have also been probed, including effects on coenzyme binding/release, thermodynamic control of electron transfer, conformational coupling between partner proteins and vectorial versus ‘off pathway’ electron flow. Here, we review these studies and discuss evidence for the emergence of dynamic structural models of electron flow along human microsomal CPR–P450 redox chains.

**Abbreviations**
CPR: cytochrome P450 reductase
CYP2C9: cytochrome P450 2C9
CYP3A4: cytochrome P450 3A4
CYP: cytochrome P450
ER: endoplasmic reticulum
FAD: flavin adenine dinucleotide
FMN: flavin mononucleotide
FRAP: fluorescence recovery after photobleaching
FRET: Förster resonance energy transfer
HO: haem oxygenase
HSQC: ^1^H,^15^N heteronuclear single quantum correlation
IM‐MS: ion mobility mass spectrometry
*k*_obs_: observed rate constant
methyl‐TROSY: methyl‐transverse relaxation optimized spectroscopy
MSR: methionine synthase reductase
NADP(H): nicotinamide adenine dinucleotide phosphate
NOS: nitric oxide synthase
PELDOR: pulsed electron–electron double resonance
RAS: reflective anisotropy spectroscopy
SANS: small‐angle neutron scattering
SAXS: small‐angle X‐ray scattering
SM: single molecule

## Introduction

The extraordinary ability of enzyme molecules to catalyse chemical reactions with rates of many orders of magnitude over uncatalysed reactions, often with high selectivity and specificity, has drawn the attention of researchers over several decades. The use of enzymes for green industrial processes, or as targets for drug interaction, are major research activities and to support these efforts, an understanding of the structural and physical basis of enzyme catalysis (i.e. nature's design rules) is required. A branch of enzymology that has gained much interest and is challenging our understanding of catalysis is the study of dynamics 1. Enzyme dynamics are involved in a broad range of physiologically important processes, including rotations of the c and ϒ subunits of ATP synthase 2, domain motions of the Rieske iron–sulphur cluster of cytochrome *bc*_1_ 3 and substrate recognition facilitated by motions of the DFG‐motif loop in adenylate kinase 4 (important in metabolic monitoring/signalling). Because protein motions occur over extensive time (ps‐s) and distance (0.1–100 Å) scales, it is often challenging to experimentally determine the influence of motions on enzyme catalytic cycles.

The study of a number of soluble model systems (e.g. dihydrofolate reductase (DHFR) 5, 6; members of the Old Yellow Enzyme (OYE) family 7, among others) have shed some light on the relationship between dynamics and catalysis. Studies with membrane‐bound enzymes, however, are more challenging, requiring the use of sophisticated biochemical and spectroscopic tools to study the influence of motions either in solubilized preparations or in the context of lipid bilayer environments. Where possible, a strategy has been one of ‘divide and conquer’, in which studies of membrane‐free (e.g. truncated) forms of an enzyme provide some insight; this can then be translated to the more challenging context of the bilayer environment. Here, we review recent advances in our understanding of a prototypical membrane‐bound system involving cytochrome P450 reductase and cognate redox partners (CYPs). We illustrate how integration of different methods from a toolbox of cutting‐edge spectroscopic and structural approaches can provide a more holistic understanding of the impact of dynamics on enzyme catalysis for membrane‐bound systems.

At the time of writing this contribution, there are over 5000 publications on the structure, function and mechanism of cytochrome P450 reductase (CPR). Supported by advances in spectroscopy, structural biology and membrane biology approaches, we are now on the cusp of understanding how protein dynamics and the membrane environment modulate the flow of electrons from NADPH, through CPR, to the superfamily of drug detoxifying cytochrome P450 (CYP) proteins. This is captured in multiple reports published in recent years on the topic of dynamics and how this relates to electron flow in multidomain proteins such as CPR. It is timely to take stock of these developments and to critically assess current knowledge. Here, we provide this overview and an evaluation of mechanistic insights gained from recently published works on the CPR enzyme family.

**Figure 1:**
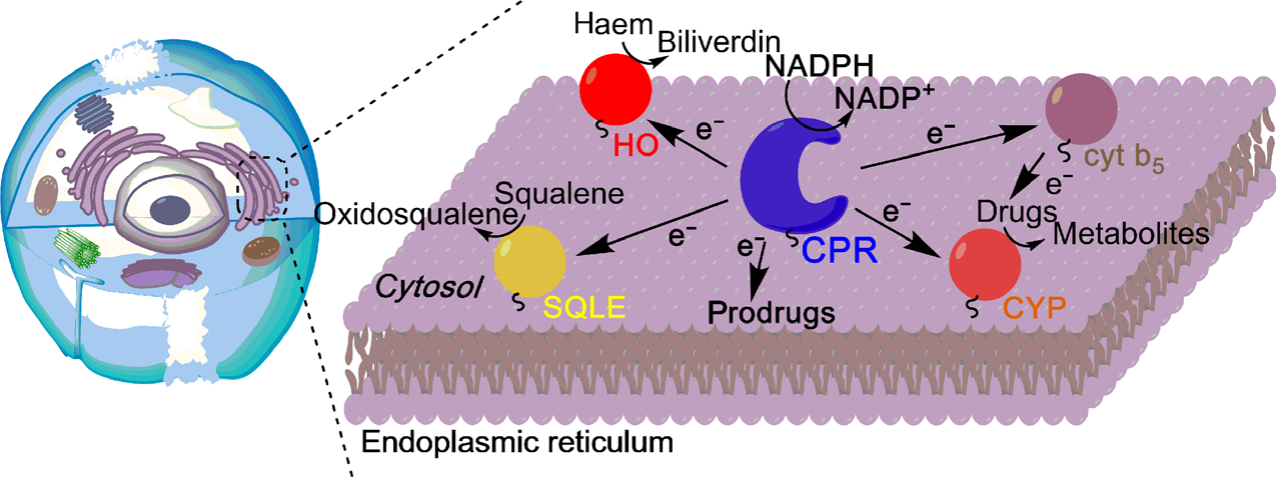
Cellular location and function of mammalian CPR. CPR is anchored to the cytosolic side of the ER, where it receives electrons from NADPH before passing them to a variety of partner proteins. The partner proteins of CPR include HO (shown as a red sphere), squalene monooxygenase (SQLE, shown as a yellow sphere), cytochrome *b*_5_ (cyt *b*_5_, shown as a brown sphere) and the superfamily of cytochrome P450 proteins (CYPs, shown as an orange sphere). CPR is also known to activate a wide variety of prodrugs, such as the anticancer drug, mitomycin C.

Cytochrome P450 reductase has proven to be an excellent protein system to study the impact of domain dynamics on electron transfer chemistry. Lessons learned not only further understanding of the relationship between dynamics and electron flow but also extend to related enzymes (i.e. the wider di‐flavin oxidoreductase family 8, 9), which include the physiologically important nitric oxide synthases (NOS) and methionine synthase reductase (MSR). In mammalian cells, CPR plays a crucial role in drug and xenobiotic metabolism. As the enzyme can be obtained in high yields and in soluble form (i.e. lacking the N‐terminal membrane anchor region) using standard expression and purification protocols 10, it has been subjected to numerous biochemical and mechanistic studies extending over many decades. Only recently, however, has the impact of domain dynamics on enzyme catalysis been investigated in detail. Interest in understanding the interplay between dynamics and biological function has been driven in part by access to improved biophysical time‐resolved spectroscopy and structural biology methods. At a basic level, CPR functions by passing electrons from the nicotinamide coenzyme NADPH (by formal hydride transfer), then sequentially to a FAD and a FMN cofactor, and ultimately to a variety of electron‐accepting partner proteins located on the surface of the endoplasmic reticulum (ER) 9. These partners include, but are not limited to, CYPs 11, haem oxygenase 12, squalene monooxygenase 13 and cytochrome *b*_5_ 14 (Fig. 1). The mechanism by which CPR transfers electrons to partner proteins is complex and evidence suggests this is achieved through conformational sampling of CPR by relative reorganization of cofactor‐binding protein domains 8, 9, 10, 15, 16, 17, 18, 19, 20, 21, 22, 23, 24, 25.

How the dynamics of CPR and its membrane environment influence electron flow and P450 catalysis has been debated since the first visualization of the ‘static’ X‐ray crystal structure of rat CPR 26. This structure raised important questions:

- Does a structural–functional landscape exist that is defined by relative CPR domain motions?
- What is the extent of this putative landscape?
- Do in‐built mechanistic trigger(s) (e.g. coenzyme binding, redox chemistry) facilitate migration across this landscape?
- Do environmental factors (e.g. membrane lipid composition) influence this landscape?
- Do physical perturbations (e.g. pressure, temperature, ionic strength, viscosity) influence this landscape and can these be used as experimental probes of the relationship between structural dynamics and function?

Cytochrome P450 reductase is located on the cytoplasmic side of the ER where, in principle, it could adopt multiple conformational states 8, 9, 10, 15, 16, 17, 18, 19, 20, 21, 22, 23, 24, 25. These would range from ‘closed’ forms of the enzyme, with relatively short distances between the flavin cofactors, to more ‘open’ states, where the flavin cofactors are more physically separated. Static crystal structures of CPR suggest that large‐scale domain motion is needed to enable the flow and delivery of electrons to partner proteins (e.g. CYPs). Intuitively, interflavin electron transfer would be optimal (i.e. maximized electronic coupling) in ‘closed’ form(s) of CPR. Conversely, interprotein electron transfer (e.g. CPR to CYP) would require a more ‘open’ state, to enable closer, transient approach of the CPR FMN cofactor to a CYP partner. These are simple models based on ‘static’ crystal structures alone and supported by *in silico* (rigid body) modelling. Missing until recently has been compelling biophysical and structural evidence for the spatial and temporal control of structural change and associated mechanistic trigger(s) to coordinate electron flow and CYP catalysis. In this review, we discuss evidence in support of a dynamic functional model for CPR and related proteins obtained from studies with solubilized CPR proteins (i.e. lacking a membrane localizing region) and native full‐length CPR in native‐like conditions (i.e. embedded in membrane nanodiscs).

## Multiple conformations in crystal structures

**Figure 2:**
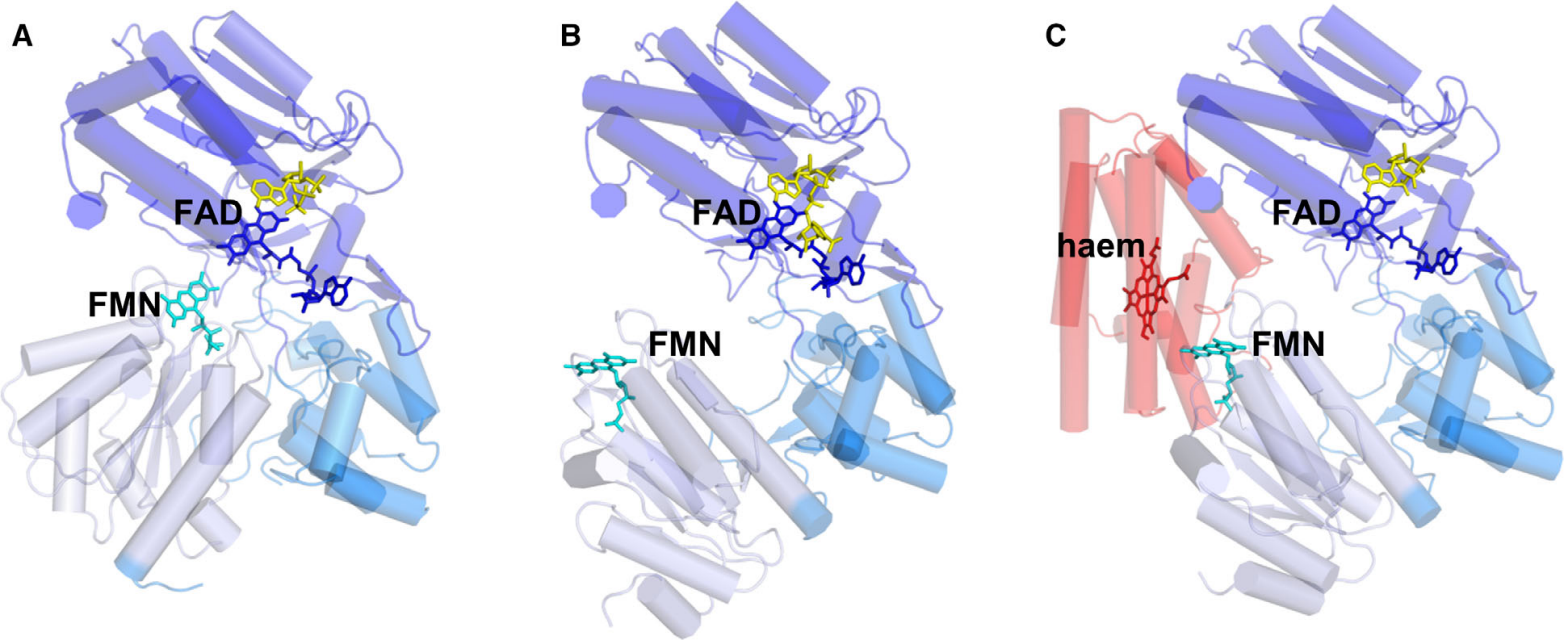
X‐ray structures of (A) ‘closed’ CPR, (PDB ID 1AMO), (B) ‘open’ (ΔTGEE) CPR (PDB ID 3ES9) and (C) HO‐bound ‘open’ (ΔTGEE) CPR (PDB ID 3WKT). The FAD‐containing, connecting, and FMN‐containing domains of CPR are shown as dark blue, marine blue and light blue cartoons respectively. HO is shown as a red cartoon. The FAD, FMN, NADP^+^ and haem cofactors are shown as dark blue, cyan, yellow and red respectively.

Structures of the soluble portions of CPR are shown in Fig. 2 26, 27, 28. Like other members of the di‐flavin oxidoreductase family, CPR contains two noncovalently bound flavin cofactors, a FAD cofactor (located in FAD‐binding ferredoxin‐NADP^+^ reductase‐like domain) and a FMN cofactor (located in an FMN‐containing ferrodoxin‐like domain). As well as these two cofactor‐binding domains, CPR contains a third structural region, which encompasses a connecting domain and a highly dynamic ~ 15 amino acid linker region 18, 26.

From a mechanistic viewpoint, the X‐ray crystallographic structure of wild‐type CPR provides valuable but limited insight into the function of the enzyme 26. This follows because the FMN cofactor is buried. How this is ‘liberated’ to connect with partner proteins (both electronically and by protein–protein interaction) is not clear from static X‐ray crystallographic structures alone 16. In this ‘closed’ conformation for wild‐type CPR (Fig. 2A), the FMN is unable to connect to CYP or other partner proteins to donate electrons. This follows because the distance between the FMN and the haem of CYP is greater than the 15‐Å limit for efficient electron transfer 29, 30, 31, and a number of important residues on the surface of CPR, which are thought to interact with a patch of basic residues on the surface of CYPs, are occluded in this ‘closed’ state 32. Also, there is an inconsistency between the observed CPR structure and transient state kinetic measurements. Based on the 4‐Å edge‐to‐edge distance between the FAD and FMN cofactors seen in the CPR structure 26, a free energy optimized interflavin electron transfer of *ca* 10^10^ s^−1^ would be expected 29. However, interflavin electron transfer in CPR is slow (10–55 s^−1^) 33, 34, 35, 36, 37. This suggests that electron transfer is ‘gated’ and controlled by conformational change 15 and/or other events (e.g. proton transfer) 38.

More recent crystallographic work on variant forms of CPR has afforded further insight from which models of electron flow are derived. These studies have led to the publication of structural data and accompanying kinetic studies for a variety of artificially ‘opened’ and ‘closed’ CPR variants. These include a disulphide locked CPR variant 27, which resembles the ‘closed’ conformation of CPR. In this ‘closed’ CPR, electron transfer to a partner protein is impaired, while interflavin electron transfer rates are maintained (albeit slower than that expected for a pure nonadiabatic electron transfer process), implying that the reaction is still controlled by conformational sampling of multiple, constrained CPR structures.

By removal of selected residues in the linker region of CPR (variant ΔTGEE CPR), the structures of once elusive ‘open’ forms of CPR have been determined (Fig. 2B) 28. Distances between the dimethyl benzene rings of FAD and FMN in the structures of ‘open’ CPR range from 29 to 60 Å (different distances were observed in each of the three structures determined for the ‘open’ state) 28. Unlike the disulphide locked ‘closed’ form, this ‘open’ variant was unable to catalyse electron transfer from FAD to FMN. However, the variant was able to catalyse interprotein electron transfer from CPR to partner CYP proteins, in line with the proposed requirement for an ‘open’ form for partner protein recognition and optimal electronic coupling of the CPR FMN and CYP haem centres. Structures for ‘open’ conformers have also been observed using other biophysical approaches. For example, an ‘open’ structure of a yeast–human chimeric CPR protein has been observed 39. In this case, there is an 84‐Å edge‐to‐edge distance between the two flavin cofactors. Also, the structure of the ΔTGEE variant of CPR in complex with haem oxygenase (HO) has been reported 40, which shows a 30‐Å distance between the FAD and FMN cofactors and a 6‐Å distance between the CPR FMN and the oxygenase haem (Fig. 2C). Taken together, and consistent with earlier predictions that emerged from ‘static’ X‐ray structures of wild‐type CPR, these studies imply that a ‘closed’ form of CPR is required for interflavin electron transfer whilst an ‘open’ form is needed for CPR‐partner protein electron transfer. These important crystallographic studies provide strong evidence to support a need for large‐scale domain motion during CPR catalysis, but they do not inform on the solution state of the enzyme, nor do they inform directly on the coupling of dynamics to function. Additional solution‐based biophysical methods are therefore required to corroborate and extend these findings.

## Binary solution structural models

Solution structural methods and time‐resolved spectroscopy have contributed to our understanding of CPR dynamics in recent years. These include the use of NMR spectroscopy, small‐angle X‐ray and neutron scattering, mass spectrometry and time‐resolved fluorescence spectrofluorimetry, the outputs of which are now being integrated into more holistic models of catalysis that have emerged from conventional stopped‐flow absorbance spectroscopy. In this section, we evaluate these studies and provide a perspective on how ‘open’ and ‘closed’ states of CPR (as a simple two‐state model) relate to flavin redox state, the binding of coenzyme and enzyme turnover.

### Nuclear magnetic resonance

Nuclear magnetic resonance offers detailed insight of the solution structure of proteins but its application is typically restricted to medium‐sized proteins (< 40 kDa). However, high‐field spectrometers and novel NMR methods are now extending these capabilities. Solution NMR studies of the CPR FMN domain 41 and full‐length and soluble CPR (70 kDa) have been reported 18, 21. Two studies with full‐length and soluble CPR show contrasting views. In a study published by Ellis *et al*. 21, the FMN domain was assigned based on ^1^H,^15^N heteronuclear single‐quantum correlation (HSQC) spectra using the corresponding spectra from the isolated CPR FMN domain and the minimum chemical shift difference approach. The authors suggested that the FMN domain exists in at least two conformational states (‘open’ and ‘closed’) that have different interaction surfaces with the FAD domain. In more recent work by Vincent *et al*. 18, the CPR backbone was sequentially assigned. The assignment was partial (60 %), but information was obtained on the dynamics of CPR, specifically through ^15^N relaxation and ^1^H‐^15^N residual dipolar coupling measurements. A region of elevated mobility in the fast timescale region (ps–ns) was identified in the linker positioned between the connecting domain and the CPR FMN domain 18. The authors concluded that this linker plays a role in stabilizing the FAD–FMN domain interface in the ‘closed’ state and may be involved in switching conformations to more ‘open’ states. The study revealed that, in the absence of nicotinamide coenzyme, the oxidized form of CPR exists in solution in a ‘closed’ state, a finding that is in excellent agreement with X‐ray crystallography 18.

The role of redox chemistry in driving CPR conformational change has been evaluated using NMR spectroscopy. Surface‐exposed cysteines were introduced into a ‘cysteine‐free’ variant of CPR by conventional protein engineering. Galiakhmetov *et al*. 25 then attached ^13^C‐labelled methyl probes to thiol groups on the surface of the enzyme and used methyl‐TROSY methods to investigate CPR dynamics. Although the affinity for NADPH and catalytic activity of the CPR variant used in this study are different to wild‐type enzyme 25, 27, a number of interesting observations emerged. Specifically, it was found that CPR adopts different states in oxidized and NADPH‐reduced forms. The reduced form of CPR takes up an ‘open’ conformation and membrane‐bound CPR adopts similar structures to that of the truncated, soluble form of CPR 25.

### Small‐angle scattering

Small‐angle neutron scattering (SANS) studies have been performed with CPR to analyse the effect of flavin redox state on CPR conformational equilibria 20, 22, 42. These studies were a reappraisal of previous work, where small‐angle X‐ray scattering (SAXS) was used to monitor redox‐dependent conformational change in solution 21. SANS offers a number of advantages over SAXS. Unlike SANS, SAXS leads to the production of *in situ* photoelectrons 43 that could reduce the flavin cofactors in CPR. SANS studies were therefore timely, as they do not suffer from this limitation. SANS led to a description of the solution conformation of CPR in the context of a two‐state (binary) model in which CPR exists as a mixed population of ‘open’ and ‘closed’ states 20, 22. The authors concluded that CPR is predominantly ‘closed’ when the flavin cofactors are oxidized and ‘open’ following reduction of the flavin cofactors. They suggested that the binding of NADP(H) to two‐ and four‐electron reduced forms of CPR leads to repopulation of the ‘closed’ state. This coenzyme binding effect on CPR conformational equilibria is consistent with transient state kinetic studies used to measure rates of interflavin electron transfer in the absence/presence of NADP(H). Altered kinetics on binding NADP(H) were interpreted to reflect perturbation of CPR solution structure 34, 44.

### Ion mobility mass spectrometry

Electrospray ionization methods in combination with ion mobility mass spectrometry (IM‐MS) have proven to be powerful in studies of protein conformational change. As a relatively low‐resolution structural approach, IM‐MS can be used to investigate large‐scale protein dynamics 45. Despite limitations (e.g. potential collapse of protein structure within picoseconds of dehydration in the gas phase 46), IM‐MS has been used to study ligand‐induced conformational change in a selection of proteins and to identify new conformations (e.g. ubiquitin 47). IM‐MS indicates that CPR is in a ‘closed’ state in a FADH_2_‐FMNH_2_ form generated by titration with NADPH under anaerobic conditions, and that oxidation of CPR leads to an increased population of a more ‘open’ state 19. IM‐MS therefore confirms that CPR exists in a dynamic equilibrium in the gas phase, similar to that proposed in solution. Moreover, IM‐MS was able to demonstrate that the relative abundance of the two detected conformations (inferred to represent ‘open’ and ‘closed’ states) is influenced also by the ionic strength of the solution that was used to prepare the enzyme sample for electrospray MS analysis.

### Fluorescence

Ensemble and single‐molecule (SM) fluorescence studies of CPR using intrinsic flavin and thiol‐linked fluorophore fluorescence have been reported. Some of this work has been reviewed with reference to di‐flavin oxidoreductases in general 48. Here, we focus briefly on studies reported in the last 5 years where fluorescence has been used to explore CPR dynamics. SM approaches are insightful as they can detect sample heterogeneity and transient events that are lost when working with ensemble‐based approaches. SM fluorescence studies of a nanodisc‐bound CPR cysteine knock‐in variant from *Sorghum bicolor* have been informative 17, and extended through the use of SM Förster resonance energy transfer (FRET). Plant CPRs may be different to their mammalian counterparts as they crystallize in ‘open’ states 49 and display different catalytic turnover kinetics 50. Notwithstanding, SM fluorescence studies have demonstrated that *S. bicolor* CPR populates at least two states in solution, one with high activity and another with low activity 17. Real‐time FRET methods have been used to demonstrate CPR conformational change during enzyme reduction by NADPH, and this has enabled structural change to be correlated with reaction chemistry 10. FRET and fluorescence polarization anisotropy have also been used to demonstrate redox‐dependent conformational changes of CPR 24. Here, fluorescence lifetime measurements have been used to probe the distance between two fluorophores attached to the surface of the CPR FAD and FMN domains, which show that CPR is in a ‘closed’ state in the oxidized form and maintains this ‘closed’ form when fully reduced 24.

## Complex multistate landscapes

So far, the multiple approaches presented above indicate that CPR exists both in solution and in crystal form in two structural states – ‘open’ and ‘closed’ conformations. This two‐state (binary) model has been used to rationalize functional behaviour but it is an oversimplification of the true solution structure of CPR as revealed using other solution biophysical approaches.

Pulsed electron–electron double resonance (PELDOR) spectroscopy can be used to measure the distance between paramagnetic species separated by 15–80 Å, and this allows the user to have access to multiple conformational states present when enzyme solutions are frozen at 80 K. PELDOR spectroscopy has been exploited to measure distances between the FAD and FMN semiquinone species in CPR and related di‐flavin oxidoreductases, including NOS and MSR 15, 51, 52. These studies have demonstrated that each of these enzymes exists as a continuum of ‘open’ and ‘closed’ states and that ligand binding remodels the conformational landscape. Specifically, the binding of nicotinamide coenzyme shifts the enzyme population to more ‘closed’ conformations. Also, the conformational distribution is perturbed substantially on binding a partner, such as calmodulin and methionine synthase activation domain to NOS and MSR respectively. These studies indicate that a simple binary model (‘open’ and ‘closed’) is not appropriate, that a multiconformational landscape is more representative and that remodelling of this landscape is realized through small‐molecule (coenzyme) and partner protein calmodulin (NOS), MS‐activation domain (MSR) and, by inference, P450 (CPR) binding. In the case of CPR and NOS, this structural complexity in solution is consistent with functional studies where perturbations in temperature, pressure, solvent dielectric and viscosity have been used to explore the functional consequences of moving across these multidimensional protein landscapes 15, 51, 52. This work has been reviewed elsewhere, and in more depth, in relation to the significance of the multiconformational landscape in electron transfer and overall enzyme function 9, 53.

Single‐molecule FRET has been used to explore the broader structural landscape of NOS. The reductase component of NOS is similar to CPR at the level of domain organization. Through the use of SM FRET, He *et al*. 54 have shown how the reductase portion of NOS explores multiple conformations. Given that (a) NOS reductase is structurally homologous to CPR and (b) there is good evidence for large‐scale conformational change, it is not surprising that both should populate multiple conformational states during the enzyme reaction cycle 48, 53, 55. This is influenced by CaM binding, an extended C‐terminal tail and an auto‐inhibitory loop, which regulate ‘opening’/’closing’ of the enzyme and the accompanying electron transfer processes associated with it 48, 53, 55. He *et al*. 54 have shown how a mixed population of ‘open’ and ‘closed’ NOS reductases are populated in solution and how calmodulin alters dramatically the NOS conformational landscape. In particular, this study suggests CaM binding causes faster interconversion between different NOS conformations and leads to an increase in the population of more ‘open’ states. This is consistent with a series of cryoelectron microscopy studies 56, 57, 58 that demonstrated multiple conformational states of NOS as well as being consistent with the general model for NOS catalysis proposed by many in the field, where calmodulin binding frees the C‐terminal tail sitting between the FAD and FMN domains, allowing the enzyme to occupy more ‘open’ states 48, 53, 55.

More recent SM FRET investigations of plant CPR 59 have also been used to expand the minimal two‐state (binary) model advanced from earlier studies on the same protein 17. These more recent SM FRET investigations indicate that the protein populates numerous conformational states in solution. Moreover, ionic strength and membrane environment affects on the distribution of distinct protein conformers across the conformational landscape was also demonstrated 59.

These emerging biophysical studies emphasize the need to incorporate multiple conformational states to describe the landscape of CPR and related di‐flavin oxidoreductases. Although conceptually straightforward, earlier two‐state models (‘open’ and ‘closed’) can now be discounted as an oversimplification of solution structure. The challenge now is to relate the conformational complexity with reaction chemistry, which we turn to in the next section.

## Correlating dynamics with the enzyme reaction cycle

To more fully understand the relationship between protein domain dynamics and CPR turnover, a description of conformational change during enzyme catalysis is required. In recent years, biophysical methods for studying dynamics relevant to catalysis have been developed for CPR and other members of the di‐flavin oxidoreductase family 10, 23, 60, 61, 62, 63. In selected cases, these have been extended to identify trigger(s) (e.g. coenzyme binding and flavin reduction) of conformational change 60. In this section, we describe the relative strengths of using ‘real‐time’ approaches to correlate dynamics with enzyme chemistry.

**Figure 3:**
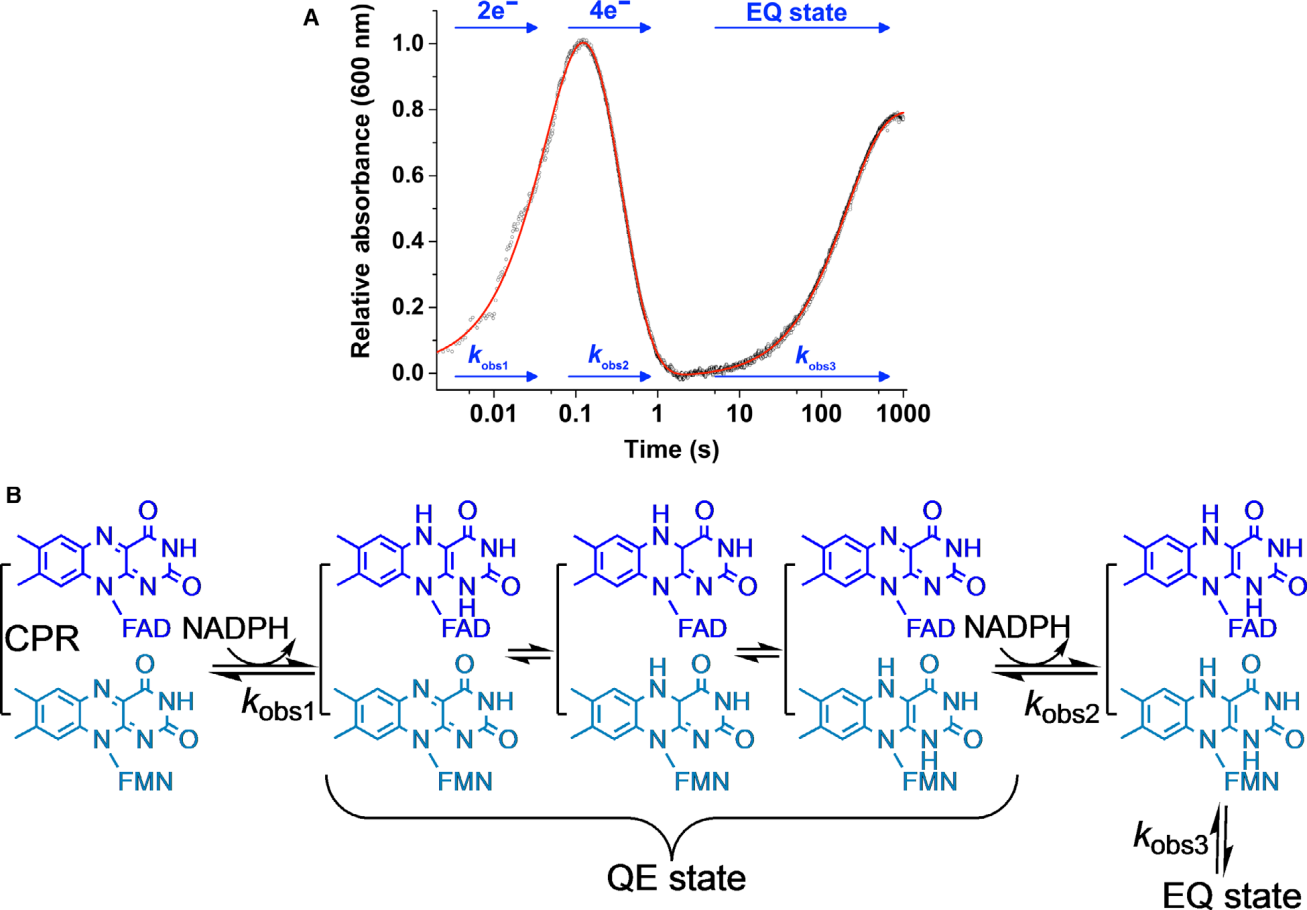
The reaction mechanism of CPR. (A) shows an example stopped‐flow transient for CPR flavin reduction. The CPR reductive half reaction comprises three kinetic phases. The first two of these kinetic phases are loosely related to two‐ and four‐electron reduction, while the third, slow phase is related to the EQ state of CPR. This EQ state is hypothesized to be a conformational change and/or further oxidation of NADPH attributed to thermodynamic relaxation. (B) shows the proposed mechanism of CPR catalysed flavin reduction. In (B) the QE state refers to the quasi‐equilibrium state of CPR, a state where electrons are distributed between the FAD and FMN cofactors.

Rapid mixing stopped‐flow absorption traces for the reaction between CPR and NADPH are complex (Fig. 3). Studies of flavin reduction in CPR are typically conducted by mixing NADPH with oxidized CPR and monitoring time‐dependent changes in the absorbance of enzyme‐bound flavin cofactor 35. Flavin reduction occurs in three kinetically resolvable steps (Fig. 3). While two of these steps are loosely related to the two‐ (*k*_obs1_) and four‐electron (*k*_obs2_) reduction in the protein (a result of two formal hydride transfer events), the third step (*k*_obs3_), which appears over long time excursions (> 100 s), is not relevant to catalytic turnover (i.e. the observed rate constant is much slower than steady‐state turnover values). The enzyme species that accumulates in this slow step is sometimes termed the ‘EQ state’, and likely represents spectral change following slow conformational change and/or further oxidation of NADPH attributed to thermodynamic relaxation 35, 60, 64, 65. Despite the well‐documented existence of this slow kinetic phase in the literature, formation of this final enzyme species following a reduction in CPR (e.g. with NADPH) has led to some confusion relating to the catalytic relevance of ‘open’ and ‘closed’ states. There are now numerous publications in which the conformational state of CPR has been analysed in different reduced states following the addition of a reductant (e.g. NADPH or dithionite). Structural analysis of these reduced forms will report on the ‘EQ’ or related slowly forming states, which cannot be mapped into the catalytic cycle. Consequently, ‘real‐time’ methods that monitor conformational change during catalysis are needed to correlate the importance of structural transitions with the reaction coordinate. In this way, the importance of ‘opening’ and ‘closure’ to individual reaction steps can be ascertained. This then provides new insight into dynamic models of catalysis. Below, we describe ‘real‐time’ methods that have been reported recently from which detailed models of CPR catalysis are beginning to emerge.

### Reflective anisotropy spectroscopy

**Figure 4:**
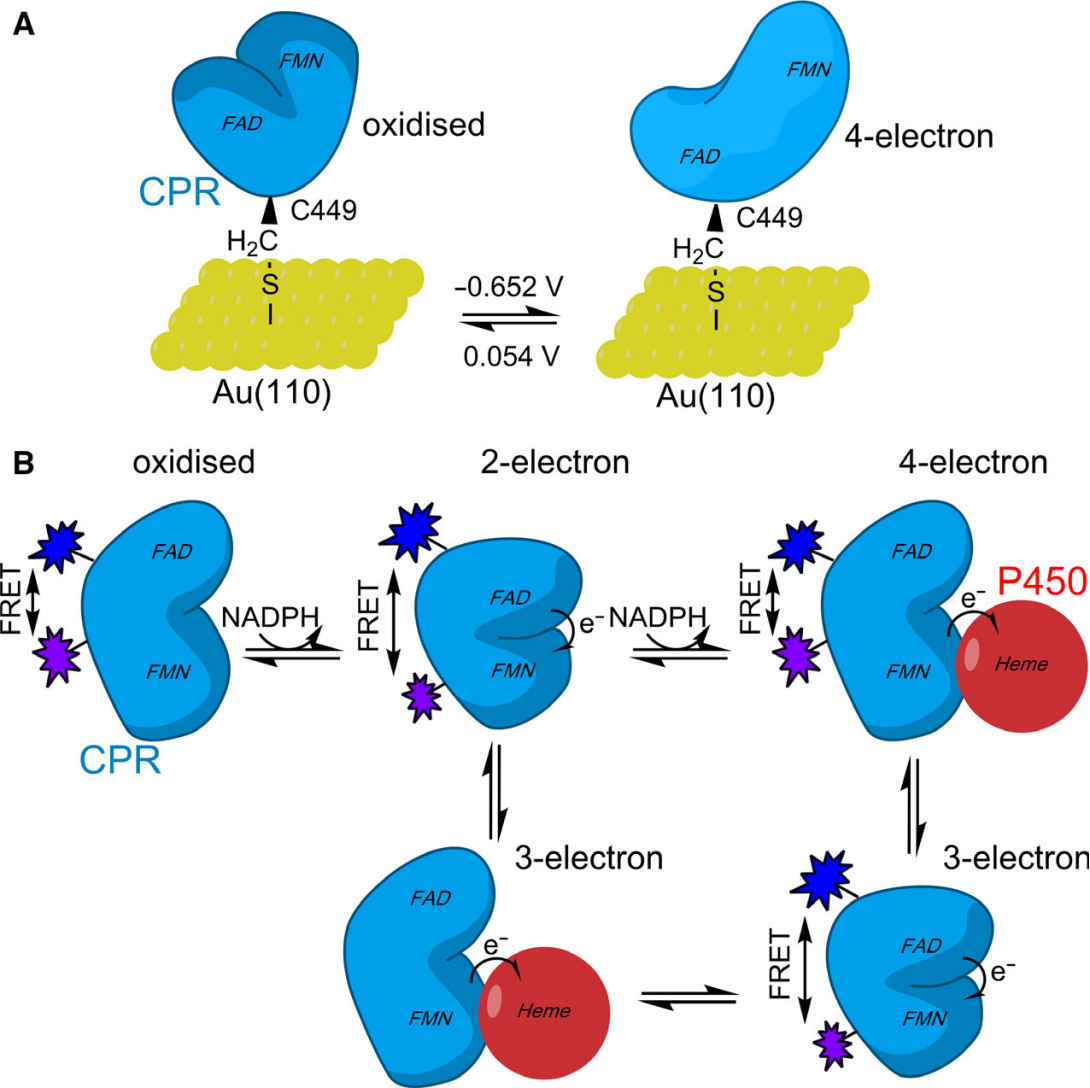
Correlating dynamics with the enzyme reaction cycle using ‘real‐time’ methods. (A) A schematic showing how RAS has been used to probe ‘real‐time’ dynamics of CPR related to redox state. (B) A schematic showing how CPR domain dynamics have been linked to the reaction coordinate through the use of FRET stopped‐flow methods. As the three‐electron reduced, ‘open’ form of CPR has not been investigated/detected by stopped‐flow FRET methods, we have omitted the fluorophores bound from this CPR conformation in (B).

Reflective anisotropy spectroscopy (RAS) is a ‘real‐time’ method that has been used to access the conformational landscape of CPR (Fig. 4A) 61, 62, 63. RAS is used to measure the difference in normal‐incidence reflectance for two different linear polarization directions 66, and has been used in recent years to monitor a conformational change in the number of complex redox enzymes that have been immobilized on gold electrodes 61, 62, 63, 67. For CPR, an exposed cysteine residue was engineered on the surface of the enzyme (CPR variant P449C), and CPR‐Au(110) assemblies prepared that were suitable for combined electrochemical and reflectance anisotropy spectroscopy (RAS). Electrochemically driven electron exchange between the Au electrode and immobilized CPR then enabled conformational change to be detected in ‘real time’ using RAS spectroscopy. RAS revealed that electrochemically driven changes in the redox state of CPR flavin cofactors is accompanied by alterations in the conformational state of the enzyme (Fig. 4A). When the protein is in an oxidized state, RAS indicates that the enzyme is in a ‘closed’ form. Conversely, when the protein is reduced to the four‐electron level (mimicking the redox state following two formal hydride transfer reactions) CPR adopts a more ‘open’ state. These observations are consistent with simple models in which CPR has to explore ‘open’ and ‘closed’ states to accommodate interflavin electron transfer partner protein binding. RAS indicates that immobilization of CPR onto the Au electrode does not impair dynamics linked to electron transfer.

### Stopped‐flow FRET

Rapid mixing stopped‐flow FRET measurements have enabled real‐time analysis of conformational change on timescales similar to catalysis. In this approach, a site‐directed fluorophore‐labelling strategy was used to decorate the enzyme with extrinsic FRET ‘donor’ and ‘acceptor’ molecules. FRET is a powerful approach for studying conformational change as any changes in the emission of the ‘donor’ and ‘acceptor’ fluorophores report on localized structural change arising from biological events, including ligand/inhibitor binding and protein–protein interaction 68. By rapidly mixing fluorophore‐decorated CPR with redox partners (e.g. NADPH or partner protein) in a stopped‐flow instrument, it has been possible to monitor CPR structural change during turnover (Fig. 4B). This has enabled correlation of chemical change (monitored by UV‐Vis stopped‐flow) with protein domain dynamics (monitored by FRET stopped‐flow), and has demonstrated that coenzyme binding alters the conformation of oxidized enzyme from a predominantly ‘open’ to ‘closed’ state 23. Moreover, the rate of reduction of CPR at the two‐electron and four‐electron level is comparable to observed rates of domain motion in transitioning, respectively, from ‘open’ to ‘closed’ and from ‘closed’ to ‘open’ states (Fig. 4B) 60. This stopped‐flow FRET approach was recently extended to investigate structural change during NOS catalysis 60. Here, the FRET labels were appended to calmodulin because the reductase component has a large number of natural cysteine residues. Notwithstanding, stopped‐flow studies demonstrated that calmodulin (and by inference the reductase component) undergoes structural change that can be correlated with reaction chemistry. In this study, the natural FMN was also substituted with 5‐deazaFMN. This non‐natural form of FMN cannot be reduced to the one‐electron level and this presents a thermodynamic block on internal electron transfer from the FAD to FMN cofactors. In stopped‐flow FRET studies of 5‐deazaFMN‐substituted NOS, it was shown that nicotinamide coenzyme binding and FAD reduction (not FMN reduction) are the triggers of structural change 60. This likely represents coenzyme‐dependent displacement of the extended C‐terminal tail from the coenzyme‐binding site, as seen in the crystal structure of neuronal NOS synthase reductase 69, 70.

Stopped‐flow FRET methods are informative and will no doubt be extended to incorporate studies with variant proteins with fluorophore labels located strategically to access the kinetics and extent of protein structural change. These assays allow direct monitoring of structural change during catalysis and enable kinetic mapping of domain motions to individual chemical steps in the enzyme reaction cycle.

## The membrane environment

The majority of mechanistic studies performed on CPR have been conducted on the soluble N‐terminal truncated version of the enzyme. This form of CPR has proved to be an excellent system to study electron transfer chemistry and domain motions but it may not be representative of the membrane‐tethered version of the protein. In the cell, CPR is anchored to the surface of the ER by a 6‐kDa helix 11, 26, 71. There are many reasons why enzymes are tethered to the membrane 72. Development of new technologies in membrane biology (e.g. nanodiscs; ER biomimetics) is now opening up studies of the importance of membrane environment in CPR function.

Nanodiscs are soluble phospholipid bilayer mimics comprising a disc‐shaped apolipoprotein A belt surrounding a lipid membrane bilayer 73, 74. Compared to traditional methods of studying membrane proteins (e.g. use of insoluble lipid vesicles), nanodiscs are advantageous as they are soluble, stable and are homogenous in size 74. CPR and many of its partner proteins (e.g. CYP and cytochrome *b*_5_) have been studied in nanodisc assemblies. Notable studies have shown how binding to the membrane and alteration of membrane lipid composition influences flavin midpoint reduction potentials 75 and how different CPR : CYP ratios influence rates of CYP catalysis 76. A recent study with a 1 : 1 CPR–CYP3A4 redox pair incorporated into single nanodiscs showed that lipid composition alters the activity and redox coupling of the microsomal P450 electron transport chains 77. The development of nanodisc technology by Sligar and colleagues 73 has laid the foundation for an exciting era of CPR research, in which the structural, kinetic and dynamic properties can be characterized in a more ‘native’ environment.

Other spectroscopic methods have made it possible to probe structural and dynamic properties of CPR in lipid vesicles. The use of solid‐state NMR by Ramamoorthy and coworkers is noteworthy 78. Solid‐state NMR provides information on the structures of insoluble, noncrystalline biomolecules, such as membrane proteins. Ramamoorthy and coworkers 78 used a truncated version of the enzyme containing the FMN domain and N‐terminal membrane anchor, but lacking the FAD domain. The N‐terminal membrane domain of CPR was shown to be a transmembrane anchor with a helical secondary structure and a 13^o^ tilt relative to the membrane (as also seen for ER‐bound cytochrome *b*_5_ and CYPs). Dynamic properties of the membrane‐anchored CPR were also revealed by solid‐state NMR. Motions localized to the N‐terminal transmembrane domain are on the slower millisecond timescale, while motions associated with the FMN domain occur on the microsecond timescale. 78.

In addition to SM fluorescence studies in nanodisc environments 17, SM fluorescence recovery after photobleaching (FRAP) has revealed structural motions of mammalian CPR embedded within a detergent‐free biomimetic of the ER membrane 79, 80. FRAP is commonly used to study the lateral diffusion of membrane proteins on two‐dimensional surfaces, and to study the association of membrane‐bound biopolymers 81. FRAP studies with membrane‐bound CPR revealed that diffusion of CPR is altered on binding and reduction by NADP(H) 79, 80. This likely reflects large‐scale conformational change (as seen in nontethered CPR preparations) to enable productive interaction with downstream redox partners.

## Summary and outlook

**Figure 5:**
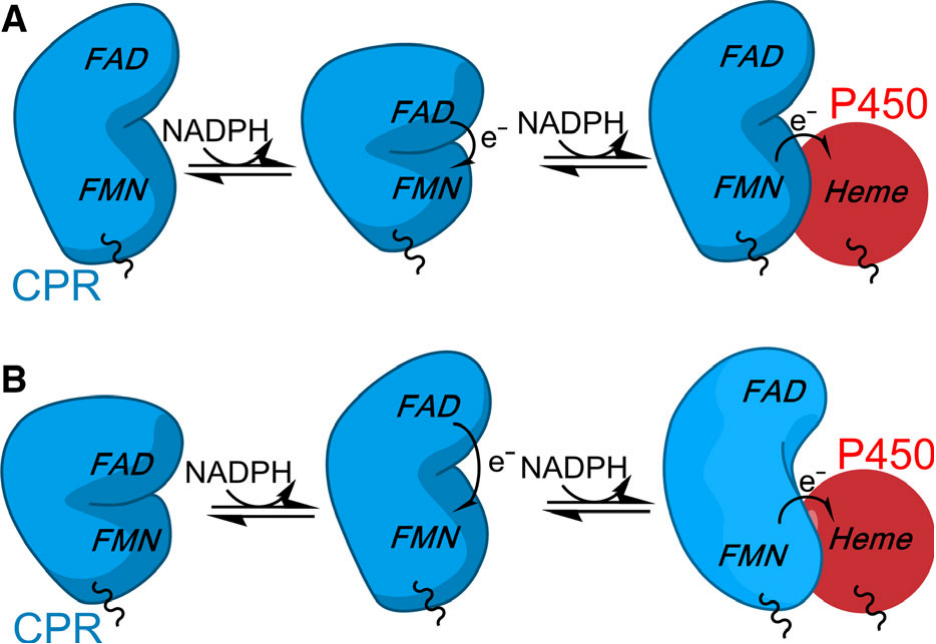
The relationship between CPR dynamics and the enzyme reaction cycle. The two models that have been proposed for CPR dynamics linked to catalysis. In the model presented at the top, CPR moves from a more ‘open’ state to a ‘closed’ state when reduced with one equivalent of NADPH. Following reduction by a second NADPH equivalent, CPR is predominantly in an ‘open’ state compatible with electron transfer to partner proteins. In the second model (below), the enzyme is ‘closed’ in the oxidized, coenzyme‐free state. Upon reduction with NADPH, CPR opens and electrons can be passed to partner proteins. It should be noted that, as CPR is purified in a one‐electron reduced state, it is often hypothesized that CPR may cycle between one‐ and three‐electron reduced states *in vivo* and not the two‐ and four‐electron reduced states portrayed here.

We are entering an exciting era for CPR research where knowledge of dynamics, especially within the membrane environment, is now accessible. This has been a long journey requiring the integration and combined use of multiple and cutting‐edge structural and (time‐resolved) biophysical methods. The approach has predominantly been one of ‘divide and conquer’, working with soluble components to establish mechanisms of electron flow from kinetic and thermodynamic perspectives and to build onto this framework a dynamic description using the methods described in this review. Despite a number of different techniques and approaches showing contrasting results, the picture that emerges (shown schematically in Fig. 5) is one in which dynamic sampling of CPR, triggered by ligand binding and redox events, facilitates electron flow from NADPH, through the internal flavin cofactors and onwards to the CYP haem cofactor at defined points in the reaction cycle to coordinate electron delivery at the point of need. Migrating these studies to more native membrane environments (e.g. nanodiscs; immobilization on electrodes) is a major challenge, but one that is now possible. What is clear, however, is that multidomain CPR and redox partners navigate a complex multiconformational landscape that is coordinated by chemical and binding events that occur throughout the enzyme catalytic cycle. This coordinated conformational change (i.e. a molecular ‘tripping the light fantastic’) ensures that electrons are delivered at the appropriate time and place during the catalytic cycle and that off pathway transfer to other proteins or acceptors (e.g. molecular oxygen) is suppressed. The importance of the CPR–CYP redox chain, and its status as a model membrane‐bound enzyme system to study dynamics linked to enzyme reaction cycles, has been a motivating factor in driving the integration of structural and (time‐resolved) biophysical approaches to the current advanced level. Studies of CPR–CYP function within the membrane bilayer are now emerging and uncovering new findings, guided by detailed insights with the solubilized component proteins. No doubt the approaches used with CPR–CYP will guide similar studies with other complex, soluble and membrane‐bound systems, as can be seen already with related diflavin reductase systems (e.g. NOS and MSR). Prospects for understanding the influence of dynamics on enzyme reaction cycles are therefore promising, including for membrane‐associated enzyme systems.

## Conflict of interest

The authors declare no conflict of interest.

## Author contributions

TMH produced an early draft of the manuscript following discussions of the content by both authors. The early draft was subsequently extended and edited by both authors to produce the final versions. TMH prepared figures.
